# Insulin amyloidosis: A case report

**DOI:** 10.3389/fmed.2023.1064832

**Published:** 2023-04-06

**Authors:** Arthur Dubernet, Maud Toulmonde, Magali Colombat, Cécile Hartog, Etienne Riviere

**Affiliations:** ^1^Internal Medicine and Infectious Diseases Unit, Haut-Lévêque Hospital, University Hospital Center of Bordeaux, Pessac, France; ^2^Oncology Unit, “Groupe Sarcome”, Bergonié Institute, Bordeaux, France; ^3^Pathology Department, Cancer University Institute of Toulouse Oncopole, University Hospital Center of Toulouse, Toulouse, France; ^4^Pathology Department, Bergonié Institute, Bordeaux, France; ^5^Faculty of Medicine, Bordeaux University, Bordeaux, France

**Keywords:** amyloidosis, insulin, type 1 diabetes, hyperglycemia, case report

## Abstract

Insulin amyloidosis is a rare form of localized amyloidosis due to insulin aggregation into subcutaneous amyloid fibrils. We describe the case of a 55 years old male with insulin-requiring type 1 diabetes presenting with two non-inflammatory intra-dermal nodules associated with local lymph node enlargement. Diagnosis was confirmed by Congo red coloration of the amyloid deposit and insulin protein identification on mass spectrometry. Insulin amyloidosis is a potential complication of repeated subcutaneous insulin injections. The main risk factor is the intrinsic characteristic of the insulin used. Insulin amyloidosis leads to systemic metabolic consequences such as chronic hyperglycemia or unpredictable hypoglycemia, as well as unesthetic cutaneous lumps or abscesses. Standard-of-care is yet to be defined but mainly rely on therapeutical education of insulin injections, while surgical excision is reported to improve glycemic control in some patients.

## Highlights

- Insulin amyloidosis (AIns) is a localized form of amyloidosis appearing in some insulin-requiring diabetic patients.
- AIns manifests as painless, non-inflammatory subcutaneous lumps, which may be overlooked as lipohypertrophy.
- In some cases, a granulomatous inflammation around the amyloid deposits can be evidenced on a 18-FDG-PET scan.
- The biopsy typically reveals amyloid deposits further characterized by immunohistochemistry, mass spectroscopy, or sequencing.
- In addition to esthetic discomfort or local infection (abscesses), AIns can lead to chronic hyperglycemia and unpredictable hypoglycemia.
- First line of care consists in patient education to avoid subsequent insulin injections in the same, or affected locations, while surgical removal has been shown to improve glycemic control in some situations.

## Introduction

Amyloidosis is a heterogenous group of diseases defined by systemic or localized amyloid fibril deposits in tissues and extracellular spaces of organs (1). Amyloid fibril is an insoluble structure resulting of a protein’s abnormal folding and aggregation in cross *β*-sheets, characterized by a green or yellow birefringence in polarization microscopy after Congo red staining. This criteria is key to retain the diagnosis of amyloidosis. In 2022, up to 42 different forms of amyloidosis were reported in the international classification established by the International Society of Amyloidosis (ISA) based on the protein contained in the amyloid deposits. Addition of a new protein to the classification implies that its whole sequence has been characterized and published (1).

Insulin’s amylogenic potential was first demonstrated in 1983 by Störkel et al. who reported the presence of amyloid deposits on a systematic skin biopsy performed in a patient treated with daily injections of porcine insulin (2). Dische confirmed the observation 5 years later and identified insulin as the component of the amyloid substance in a patient by using protein sequencing (3). Insulin amyloidosis is now recognized as a local form of amyloidosis of iatrogenic origin, abbreviated to AIns amyloidosis in the international nomenclature (1). Insulin amyloidosis is a rare, unrecognized and probably under-diagnosed condition due to its mostly asymptomatic nature (4). However, it may have clinical significance in some situations. We report the case of a 55 years old patient with insulin amyloidosis of the left arm in the context of persistent chronic hyperglycemia.

## Case description

A 55-year-old man initially presented to oncology and internal medicine consultation for several subcutaneous masses that progressively appeared on his left arm during spring 2021. Employed in construction and green spaces, his main medical history was type 1 diabetes treated by basal-bolus insulin therapy (64 IU/day at the time of the medical record), hypercholesterolemia treated with rosuvastatin, and active alcohol and tobacco consumption. He also received aspirin 160 mg daily in a primary prevention of cardiovascular events.

The patient’s type 1 diabetes was diagnosed at the age of 26 and was characterized by anti-GAD antibody positivity, poor glycemic control (8.6% of glycated hemoglobin in early 2022, 7,8-8,8% during the last 10 years) and an absence of ocular, renal, neurological or cardiovascular complication detected at the term of the last check-up in 2022. Regarding his history of diabetes treatment, he had been following a basal-bolus insulin therapy since the time of diagnosis, comprising insulin glargine as a long-acting insulin, and various types of rapid-acting insulin (human insulin initially for 16 years, insulin asparte for 10 years then insulin lispro subsequently for the last 3 years).

**Figure 1:**
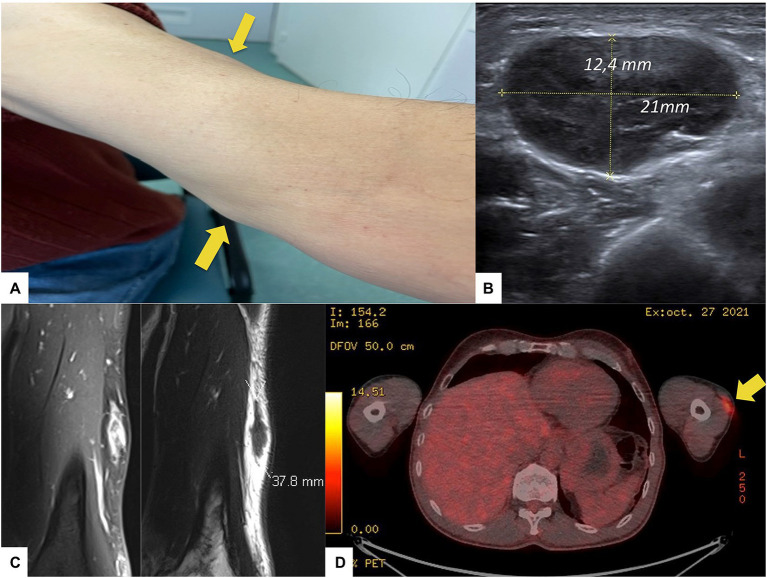
Clinical and paraclinical features of the patient. **(A)** Presence of two subcutaneous nodules on the left arm (arrows), **(B)** Soft tissue ultrasound of the left arm evidencing and measuring one subcutaneous mass, **(C)** MRI frontal slices of the left upper limb (T1 with gadolinium injection on the left, and T2 on the right), and **(D)** 18-FDG-PET-CT scan showing a 18-FDG uptake by the nodule (arrow) on the left arm.

The initial clinical examination revealed two non-inflammatory mobile, firm but painless, subcutaneous nodules measuring 2 and 5 cm on the outer and inner sides of the left forearm (Figure 1A). Two lymph node enlargements were palpable on the elbow and the left armpit, both mobile and painless. Patient’s general condition was normal, and there was no fever or other symptoms. There was no history of recent travel and neither infectious nor animal contact.

Biologically, there was no inflammatory syndrome, no renal failure, no elevation of troponin nor NT-proBNP, no abnormal blood count, no disturbance of hemostasis or transaminases, but isolated cholestasis with gamma-GT at 145 IU/l (3-ULN). There was no proteinuria, plasma protein electrophoresis showed normal levels of gamma globulins at 8.9 g/l with polyclonal shape, serum and urine free light chains and angiotensin converting enzyme were normal. Immunophenotyping of circulating lymphocytes only revealed an increase in the CD4/CD8 ratio with no argument for a lymphoid hemopathy. Broad infectious tests ruled out an infection.

**Figure 2:**
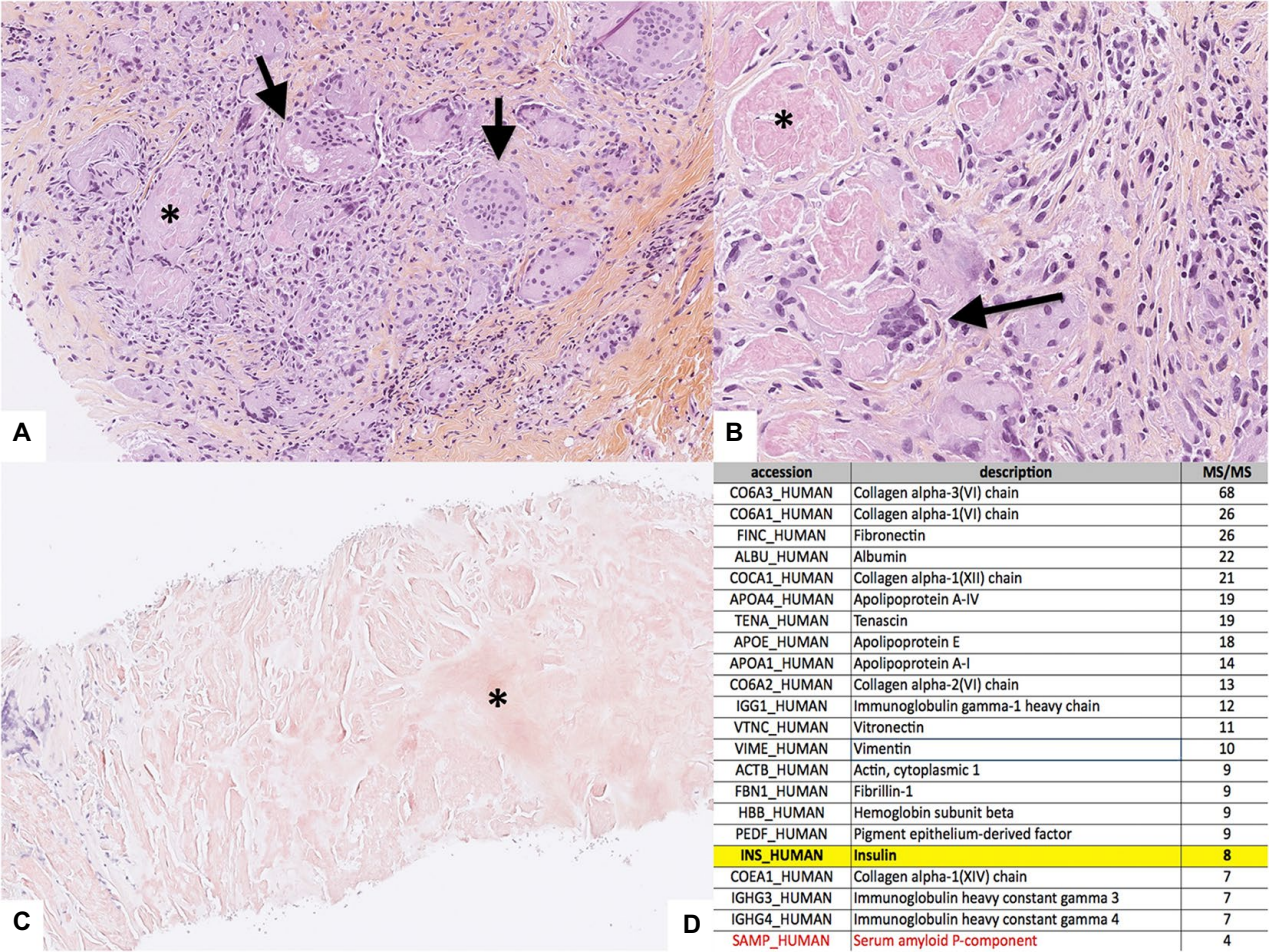
Histological analyses of the biopsy of one subcutaneous nodule. **(A)** HES section at low magnification (x10) evidencing granulomas with giant cells (arrows) phagocytizing the amorphous substance (*), **(B)** HES section at higher magnification (x40), **(C)** Section at low magnification (x4) stained with Congo Red evidencing the amyloid deposit (*), and **(D)** Detailed results of the mass spectrometry evidencing the insulin protein highlighted in yellow, along with the serum amyloid P-component in red (of note, immunoglobulin heavy gamma chains were of polyclonal origin).

A soft tissue ultrasound (Figure 1B) confirmed the two sub-cutaneous lesions of the left forearm, the main one measuring 4 cm in its longitudinal axe, associated with four enlarged lymph nodes located around elbow and armpit. An MRI of the left upper limb revealed the two sub-cutaneous lesions characterized by T2 hyposignal and enhancement upon gadolinium (Figure 1C). A 18-FDG-PET-CT-scan showed an isolated hypermetabolism (Figure 1D) colocalizing with the two subcutaneous masses and the enlarged local lymph nodes. The surgical biopsy of one lesion revealed granulomatous remodeling in contact with amyloid deposits (Figures 2A,B), displaying red-green birefringence under polarized light after Congo red staining (Figure 2C). Bacteriological and mycobacteriological cultures of the surgical specimen were both negative. Proteomic analyses using mass spectrometry confirmed the insulin origin of the amyloid deposits leading to the diagnosis of insulin amyloidosis (Figure 2D). Upon further medical history taking, the patient revealed using these two locations (among others) as injections sites for insulin.

## Discussion

Insulin amyloidosis is a rare and probably under-diagnosed condition due to its mostly asymptomatic nature (4). However, it may have clinical significance in some situations, like in our patient, with persistent chronic hyperglycemia. Indeed, AIns is a rare complication of insulin therapy in diabetic patients, mainly type 1 diabetes (66% of reported cases, like our patient), with a history of diabetes ranging from 7 to 60 years (4). Insulin is a 51-amino-acid polypeptide made of one α-chain and one β-chain linked by disulfide bridges. Although the α-chain also exhibits a propensity to aggregate, the β insulin chain is the one chain mostly involved in β cross-sheet assembly, under the influence of several factors specific to the therapeutic formulation of insulin: pH of the solution (an acidic pH promotes aggregation), high insulin concentration, elevated shear forces exerted in insulin pens or pumps, adjuvant molecules used in the formulations (e.g., silicone oil), and even the type of the insulin itself, as human insulin appear to more likely aggregate into sheets than insulin of porcine origin (4–7). Regarding analogs, they all have been reported susceptible to form amyloid fibrils, especially insulin lispro according to the work by Woods et al. (6) These five factors combined with the wide use of insulin pens and pumps since the late 1980s may partly explain the increasing number of reported cases (4). Another explanation could be a closer monitoring of insulin injection sites in diabetic patients and thus more frequent diagnoses of AIns amyloidosis (7). In the medical case we herein report, the patient had been treated with pens of rapid-acting human insulin for approximately 16 years upon diagnosis of type 1 diabetes before being treated with insulin analogs (insulin asparte first, then insulin lispro) for another 13 years, along with insulin glargine for the whole duration of the disease. Insulin amyloidosis subcutaneous nodules appeared 29 years after the disease’s onset, at a time when insulin lispro was being used as the rapid-acting insulin.

Classified as a local form of amyloidosis, AIns amyloidosis mainly manifests as firm subcutaneous nodules or masses at one or more insulin injection sites (5). Most of reported cases describe a local impact ranging from simple esthetic discomfort to infectious complications and abscesses (3). Association with lymph node enlargement at lymphatic drainage sites, as described in our case, is however uncommon: only one other case reported amyloid deposits associated with a regional adenopathy (5). Nevertheless, AIns amyloidosis can lead to more systemic symptoms, as it is often associated with metabolic complications such as hypoglycemia, or more frequently, chronic hyperglycemia. In a recent literature review, Nilsson described the difficult control of diabetes in patients with AIns amyloidosis, with a mean glycated hemoglobin of 9.7% (4). Indeed, diabetic patients with amyloid nodules prefer injecting insulin in these amyloid sites because so injections are less painful, while it is now clear that insulin absorption is much lower at these sites, around 34% of the usual absorption (6, 7). Several hypotheses have been raised: mechanical obstruction to injected insulin, or local enzymatic degradation or conversion of injected insulin into monomers by the amyloid fibrils (4). On the other hand, hypoglycemia is also frequently observed in these patients, possibly due to an unpredictable release of the insulin accumulated at the injection site(s) (4).

Like for other forms of amyloidosis, histological diagnosis of AIns amyloidosis relies on the proof of amyloid deposition using Congo red staining and characterization of the amyloid deposit using immunochemistry, mass spectrometry, amino acid analysis or sequencing (4). However, as AIns amyloidosis is a rare and poorly known condition manifesting with non-specific clinical and biological features, clinicians should rule out differential diagnoses, such as multiple myeloma, systemic amyloidosis, and other malignancies. Indeed, the occurrence of a subcutaneous mass in insulin-requiring diabetic patients is a frequent issue affecting 27 to 64.4% of these patients (8). Lipohypertrophy is the main differential diagnosis, manifesting as painless subcutaneous masses at the insulin injection site(s). In this context, the biopsy is discriminating, as lipohypertrophy is related to adipocyte hypertrophy and not insulin amyloid deposit with granulomatous reaction (5). Nonetheless, to avoid systematic skin biopsy, some authors emphasize the interest of imaging to differentiate insulin amyloidosis from lipohypertrophy, the latter distinguishing on MRI by a fat-like signal, while in our patient AIns amyloidosis displayed a gadolinium-enhanced T2 hypointense lesion (7). However, MRI characteristics of insulin amyloidosis remains scarcely known. In the work by Nagase *et al*, insulin amyloidosis seems to induce T1 hyposignal, although no details were given about T2 features and gadolinium enhancement in this report (9). In addition, we report an 18-FDG uptake by insulin amyloidosis nodules. This is interesting because amyloid deposits are mostly acellular, being composed of aggregates of misfolded proteins that are likely to induce granulomatous inflammatory reaction to resorb it. Another work by Albert *et al* reported the presence of a granulomatous reaction around the subcutaneous amyloid deposit in the skin of a 59-year old patient with severe insulin resistance (10). We believe that 18-FDG uptake is mostly due to the granulomatous inflammation, as observed in histological sections of patient’s lesions, possibly combined with high local insulin concentrations resulting in increased glucose uptake by immune and stromal cells.

Apart from lipohypertrophy, it is important to bear in mind the possibility of other causes of subcutaneous mass in patients with diabetes, such as skin cancer, lymphoma, or local infection (*staphylococcus* sp.*, streptococcus sp., mycobacterium sp., fungi*). Insulin resistance syndrome characterized by insufficient effect of subcutaneous insulin compared to intravenous in diabetic patient must also be considered (11). Of note, Soudan *et al* reported a high dermal insulin concentration in these patients, but Congo red staining seeking amyloid deposit was not performed suggesting that a fraction of these patients might rather have had AIns amyloidosis (11). Other important differential diagnoses include more common forms of amyloidosis such as AL amyloidosis, AA amyloidosis or ATTR amyloidosis. In our case, the positive diagnosis of AIns amyloidosis was made after the surgical biopsy showed a typical birefringence with Congo red staining, along with the evidence of insulin deposition on mass spectrometry. Regarding differential diagnoses, clinical, biological, radiological and histopathological findings were inconsistent with lipohypertrophy (the biopsy showed no adipocyte hypertrophy), other forms of amyloidosis (no light chain elevation, no monoclonal spike, no past or present chronic inflammation, or other amyloidogenic protein on mass spectrometry), malignancy (no other hypermetabolic location on 18-FDG PET-CT) or infection (blood, bacteriological and mycobacteriological cultures of the biopsy were negative).

Finally, as many rare presentations of common diseases, management of AIns amyloidosis is not standardized. Therapeutic education on insulin injections seems a legitimate first-line treatment so that insulin injections can be carried out in other sites and preferentially in unaffected areas, at a lower dosage and under close blood sugar control to avoid hypoglycemia. This strategy may be sufficient to improve glycaemia in most patients, as elicited in a case reported by Nagase et al. where the switch of insulin injections sites alone could reduce insulin dosage by 47% in a patient (9, 11). Surgical removal can be another treatment option, although its indications remain to be better defined. In some cases, it may improve glycemic control (12). Thus, severe and/or refractory insulin resistance despite therapeutic education to insulin injections could be potential indications for surgical removal of the nodules.

In conclusion, AIns amyloidosis remains an incompletely understood complication of insulin treatment in patients living with diabetes due to the low number of reported cases. Its real incidence and prevalence in insulin-requiring diabetic subjects is probably underestimated and under-diagnosed. This case description aims at providing physicians with a better understanding of this disease to potentially lead to a better recognition and treatment.

## Data availability statement

The raw data supporting the conclusions of this article will be made available by the authors, without undue reservation.

## Ethics statement

Written informed consent was obtained from the individual(s) for the publication of any potentially identifiable images or data included in this article.

## Author contributions

AD was responsible for formulating writing ideas and writing the manuscript. AD, MT, MC, CH, and ER were responsible for collecting information. ER was responsible for revising the manuscript and guiding the writing of the manuscript. All authors contributed to the article and approved the submitted version.

## Funding

This work was supported by Bordeaux University Hospital.

## Conflict of interest

The authors declare that the research was conducted in the absence of any commercial or financial relationships that could be construed as a potential conflict of interest

## Publisher’s note

All claims expressed in this article are solely those of the authors and do not necessarily represent those of their affiliated organizations, or those of the publisher, the editors and the reviewers. Any product that may be evaluated in this article, or claim that may be made by its manufacturer, is not guaranteed or endorsed by the publisher.
